# Antimicrobial Activity and Immunomodulatory Properties of Acidocin A, the Pediocin-like Bacteriocin with the Non-Canonical Structure

**DOI:** 10.3390/membranes12121253

**Published:** 2022-12-11

**Authors:** Daria V. Antoshina, Sergey V. Balandin, Ivan V. Bogdanov, Maria A. Vershinina, Elvira V. Sheremeteva, Ilia Yu. Toropygin, Ekaterina I. Finkina, Tatiana V. Ovchinnikova

**Affiliations:** 1 M.M. Shemyakin and Yu.A. Ovchinnikov Institute of Bioorganic Chemistry, Russian Academy of Sciences, 117997 Moscow, Russia; 2 V.N. Orekhovich Research Institute of Biomedical Chemistry, 119121 Moscow, Russia; 3 Moscow Institute of Physics and Technology, 141700 Dolgoprudny, Russia; 4 Department of Bioorganic Chemistry, Faculty of Biology, Lomonosov Moscow State University, 119234 Moscow, Russia

**Keywords:** antimicrobial peptides, bacteriocins, cytokines, lactic acid bacteria, membrane-targeting activity, multiplex xMAP assay, pediocin box, structure–activity relationship

## Abstract

Pediocin-like bacteriocins are among the natural antimicrobial agents attracting attention as scaffolds for the development of a new generation of antibiotics. Acidocin A has significant structural differences from most other members of this subclass. We studied its antibacterial and cytotoxic activity, as well as effects on the permeability of *E. coli* membranes in comparison with avicin A, the typical pediocin-like bacteriocin. Acidocin A had a more marked tendency to form an alpha-helical structure upon contact with detergent micelles, as was shown by CD spectroscopy, and demonstrated considerably less specific mode of action: it inhibited growth of Gram-positive and Gram-negative strains, which were unsusceptible to avicin A, and disrupted the integrity of outer and inner membranes of *E. coli*. However, the peptide retained a low toxicity towards normal and tumor human cells. The effect of mutations in the pediocin box of acidocin A (on average, a 2–4-fold decrease in activity) was less pronounced than is usually observed for such peptides. Using multiplex analysis, we showed that acidocin A and avicin A modulated the expression level of a number of cytokines and growth factors in primary human monocytes. Acidocin A induced the production of a number of inflammatory mediators (IL-6, TNFα, MIG/CXCL9, MCP-1/CCL2, MCP-3/CCL7, and MIP-1β) and inhibited the production of some anti-inflammatory factors (IL-1RA, MDC/CCL22). We assumed that the activity of acidocin A and similar peptides produced by lactic acid bacteria might affect the functional state of the human intestinal tract, not only through direct inhibition of various groups of symbiotic and pathogenic bacteria, but also via immunomodulatory effects.

## 1. Introduction

Pediocin-like bacteriocins (PLBs) are a large subclass of unmodified ribosomally-synthesized bacterial antimicrobial peptides that are characterized by the presence of a conserved N-terminal “YGNG(V/L)” motif (pediocin box), usually followed by “xCxxxxC” region forming a 6-residue ring [1,2,3,4]. PLBs have narrow activity spectra, with each individual peptide typically being active at submicromolar concentrations against *Listeria* spp. and a specific subset of other Gram-positive bacteria—mostly those that are closely related to the producer strain. Some of them inhibit such pathogens as vancomycin-resistant strains of *Enterococci* (VRE) [5,6]. This activity is explained by the specific binding to the mannose phosphotransferase transmembrane protein complex (Man-PTS) involved in the transport and metabolism of carbohydrates in bacteria [7]. High antimicrobial activity against a number of important pathogens, combined with low toxicity, makes PLBs attractive candidates for developing biopreservatives for the food industry and novel antibiotics for use in human and veterinary medicine [8,9]. Many natural producers of PLBs belong to the group of lactic acid bacteria (LAB), some of which have “Generally Recognized As Safe” status (GRAS) and can be used as microbial cell factories in biotechnology and also as probiotics [9,10].

**Figure 1:**
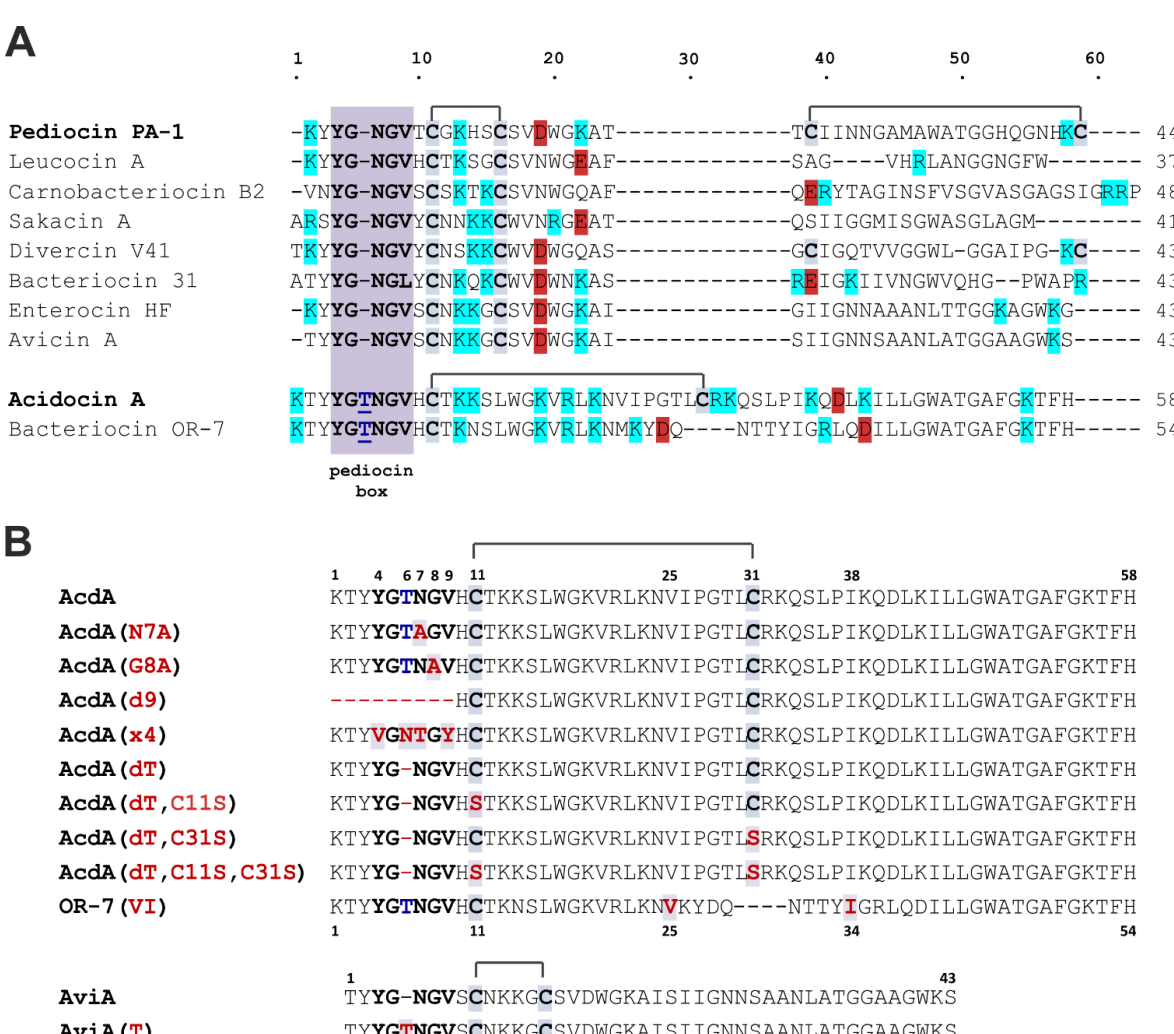
(**A**) Sequence alignment of acidocin A and the main representatives of the subfamilies of PLBs. Conservative motif YGNGV/L (pediocin box) is highlighted with purple. Cys residues that form disulfide bonds are highlighted with gray. The additional Thr residue in the “non-canonical” pediocin box of acidocin A and bacteriocin OR-7 is underlined. Positively charged Lys/Arg and negatively charged Asp/Glu residues are highlighted with bright blue and bright red, respectively. (**B**) Sequences of acidocin A (AcdA), bacteriocin OR-7(VI), avicin A (AviA), and their variants obtained in this work. The substituting/inserted amino acid residues are shown in red.

Isolation of the plasmid-encoded PLB acidocin A from *Lactobacillus acidophilus* strain TK9201, which is a starter organism for the production of fermented milk, has been first reported in 1995 [11]. Acidocin A has been shown to inhibit growth of selected species of LAB, food spoilage bacteria, and food-borne pathogens, including *Listeria monocytogenes*. This bacteriocin differs from most other representatives of the subclass by its longer amino acid sequence (58 a.a.), the size of the intramolecular ring (21 a.a.), the high positive charge of the molecule, and the non-canonical structure of the pediocin box that contains an additional Thr residue (Figure 1A). The above features should presumably affect the spectrum of antimicrobial activity and the mechanism of action of this bacteriocin; however, no information has appeared on this subject since the first publication to date. The only close homologue of acidocin A known from the literature is bacteriocin OR-7 from *Ligilactobacillus salivarius* (previously assigned to the genus *Lactobacillus*) strain NRRL B-30514, information on which is also limited [12].

The aim of this work was to elucidate whether the biological activity of acidocin A differs from that of PLBs having a “classical” type of structure. Here, we studied the antimicrobial specificity, membranolytic activity, cytotoxicity, and immunomodulatory properties of acidocin A, comparing it with other peptides and, first of all, with avicin A from *Enterococcus avium*, a representative of the classical PLBs, which is a close homologue of several well-studied bacteriocins, such as sakacin P and enterocin HF [13]. Significant differences were found in the spectra of activity and action on membranes, bringing acidocin A closer to low-selective membranolytic peptides. Acidocin A retains a relatively low toxicity to human cells and induces a number of pro-inflammatory cytokines and growth factors.

## 2. Materials and Methods

### 2.1. Antimicrobial Peptide Preparations

All peptides for this work, with the exception of melittin (Table S1), were obtained using heterologous expression in *E. coli* BL21 (DE3) as thioredoxin fusion proteins and purified by immobilized metal affinity chromatography (IMAC), CNBr cleavage, and reversed-phase high-performance liquid chromatography (RP-HPLC). The recombinant avicin A was purified as described earlier [14]. The amino acid sequences of acidocin A (GenBank acc. BAA07120.1) and the analogue of bacteriocin OR-7 [12] with M25V and M34I substitutions were reverse translated using codon optimization tool [15], according to *E. coli* codon usage bias. Coding DNA sequences (CDS) for bacteriocins were synthesized by PCR and inserted into pET-His8-TrxL vector derived from pET-His8-TrxL-Aur [16] downstream of the thioredoxin A (M37L) CDS by in vivo homology recombination [17]. Plasmids encoding acidocin A and avicin A mutants were obtained by site-directed mutagenesis using full-length plasmid amplification by inverse PCR with mutagenic primers and recircularization by in vivo homology recombination.

The transformed *E. coli* BL21 (DE3) cells were grown up to OD_600_ 0.8–1.0 at 37 °C in lysogeny broth (LB) containing 20 mM glucose, 1 mM MgSO_4_, and 100 mg/mL of ampicillin and then induced with 0.2 mM isopropyl β-D-1-thiogalactopyranoside (IPTG). The cells were cultivated in Erlenmeyer flasks for 4–6 h at 30 °C with shaking at 220 rpm, pelleted by centrifugation and sonicated in IMAC loading buffer (pH 7.8) containing 100 mM sodium phosphate, 20 mM imidazole, and 6 M guanidine hydrochloride. The clarified lysates were applied onto a column packed with Ni Sepharose 6 Fast Flow (Cytiva Life Sciences, Marlborough, MA, USA). The recombinant proteins were eluted with the buffer of the same composition supplemented with 0.5 M imidazole. The eluate fractions containing fusion proteins were acidified to pH ~1.0 (by indicator paper) and cleaved by 100-fold molar excess of cyanogen bromide over methionine for 20 h at 25 °C in the dark. The reaction products were lyophilized, dissolved in water, titrated to pH ~5.0, and loaded onto a semi-preparative Reprosil-pur C18-AQ column (250 mm × 10 mm, particle size 5 µm, pore size 120 Å) (Dr. Maisch GmbH, Ammerbuch-Entringen, Germany). RP-HPLC was performed at a flow rate of 2 mL/min in a linear gradient from 5 to 80% (*v*/*v*) of acetonitrile in water with the addition of 0.1% trifluoroacetic acid (TFA) for 58 min (Figure S1A). The peaks were monitored at 214 and 280 nm. The collected fractions were analyzed by MALDI-TOF mass spectrometry using Reflex III instrument and flexAnalysis 3 software (Bruker Daltonics GmbH & Co. KG, Bremen, Germany) and by Tris-tricine SDS-PAGE. The fractions containing the target peptides were lyophilized and dissolved in water. The peptide concentrations were estimated using UV absorbance at 280 nm.

The intramolecular disulfide bond formation in the recombinant acidocin A was confirmed using alkylation with iodoacetamide (IAA) (Sigma-Aldrich, St. Louis, MO, USA) [18]. Two peptide aliquots (30 μM in 95 μL of 100 mM potassium phosphate buffer, pH 7.8), one of which was supplemented with 2 mM dithiothreitol (DTT), were incubated at 55 °C for 30 min. Then, 5 μL of a freshly prepared 400 mM aqueous IAA solution was added to both tubes, and the samples were incubated at room temperature in the dark for another 30 min. After that, 100 μL of IMAC loading buffer was added to solubilize the peptide fraction that formed precipitate. The samples were desalted using ZipTip-C_18_ pipette tips (Merck-Millipore, Burlington, MA, USA) and analyzed by MALDI-TOF mass spectrometry.

To remove lipopolysaccharide (LPS) traces from avicin A and acidocin A samples used in cytotoxicity and cytokine response assays [19], and to remove His8-thioredoxin admixture from bacteriocin OR-7(VI) preparations, the repeated purification on a separate Reprosil-pur C18-AQ column was performed under the same conditions as above (Figure S1B). To determine LPS level, *Limulus* amebocyte lysate (LAL) test was performed with E-TOXATE kit (Sigma-Aldrich, St. Louis, MO, USA).

Melittin (>98% pure) was synthesized using a standard solid-phase method [20] in M.M. Shemyakin and Yu.A. Ovchinnikov Institute of Bioorganic Chemistry of the Russian Academy of Sciences (Moscow, Russia). The strategy of the peptide synthesis was based on the Fmoc-protocol with the TBTU/DIEA activation using Wang resin as the solid phase. The peptide was purified by RP-HPLC on Reprosil-pur C1-AQ column in the gradient of acetonitrile and analyzed by MALDI-TOF mass spectrometry, as described above.

### 2.2. Circular Dichroism Spectroscopy

Circular dichroism spectra of acidocin A in water solution and in detergent micelles of 20 mM dodecylphosphocholine (DPC) (Anatrace, Maumee, OH, USA) and 20 mM sodium dodecyl sulfate (SDS) (Sigma-Aldrich, St. Louis, MO, USA) were recorded using a J-810 spectropolarimeter (Jasco, Hachioji, Tokyo, Japan) at 25 °C in a 0.1 cm path length quartz cell (Hellma GmbH and Co. KG, Mullheim, Germany) in the 190–250 nm range [21,22]. Final concentration of the peptide was 200 μM. Four consecutive scans were performed and averaged, followed by subtraction of the blank spectrum of the solvent. The CONTINLL program with SMP56 set of reference spectra was used for data analysis.

### 2.3. Antibacterial Activity Assay

Antibacterial activity of recombinant bacteriocins against a number of Gram-positive and Gram-negative bacteria (Table S2) was determined by the method of two-fold serial dilutions in a liquid medium. Two-fold serial dilutions of bacteriocins were prepared in sterile 96-well flat-bottom polystyrene microplates (Eppendorf, Hamburg, Germany) (Cat. No. 0030730.011) in a volume of 50 µL of sterile 0.1% bovine serum albumin (BSA) (SERVA Electrophoresis GmbH, Heidelberg, Germany) to prevent non-specific sorption of peptides to the surface of the plate wells [23]. Mid-log phase bacterial cultures were diluted with the 2x Mueller-Hinton broth (MHB) (Sigma-Aldrich, St. Louis, MO, USA) to a final cell concentration of 10^6^ colony forming units (CFU) per mL. To determine the activity of bacteriocins against *Listeria* and *Enterococci*, 3% tryptic soy broth (TSB) (Merck-Millipore, Burlington, MA, USA) was used instead of MHB. Aliquots of 50 μL of diluted bacterial suspensions were added to each well of the plate, which was then incubated at 37 °C and 950 rpm for 24 h using thermostatable plate shaker PST-60HL-4 (Biosan, Riga, Latvia). As a negative control, 50 µL of the sterile growth medium (2xMHB or 6% TSB) was added to the wells containing 50 µL of 0.1% BSA. The values of the minimum inhibitory concentrations (MIC) were determined as the minimum concentration of the peptide, at which there was no visible growth of bacterial culture. Incubation for 2 h with 20 µg/mL chromogenic substrate resazurin (Sigma-Aldrich, St. Louis, MO, USA) was used to detect viable bacterial cells if they were difficult to visually detect. To study the effect of inorganic salts on the antimicrobial activity of bacteriocins, antibacterial activity assay was carried out in MHB medium supplemented with 0.9% NaCl.

Additionally, the activity of bacteriocins against *L. monocytogenes* EGD was qualitatively determined using agar diffusion (“spot-on-lawn”) assay [24]. An amount of 5 µL of water solutions containing 1–10 µg of purified bacteriocins were applied to agar plates containing 3% TSB medium with mid-log phase culture of *L. monocytogenes* EGD diluted to a OD_600_ of about 0.001 (≅7 × 10^5^ CFU/mL) and incubated overnight at 37 °C for 24 h. Antibiotic tetracycline (0.5 µg) was used as a positive control. The formation of visible zones of bacterial growth inhibition in places where bacteriocins were added indicated the presence of antimicrobial activity.

### 2.4. Selection of the Resistant Strains

A previously published protocol was used to obtain resistant forms of initially susceptible strains [25]. At the beginning of the experiment, overnight cultures of wild-type *E. coli* XDR CI 1057 and *B. licheniformis* B-511, grown to the stationary phase, were diluted with 2xMHB to prepare a bacterial suspension with a concentration of 10^6^ CFU/mL. The experiment was performed in flat-bottom 96-well microplates: 50 μL of the bacterial suspension was added to 50 μL of two-fold serial dilutions of acidocin A in 0.1% BSA to obtain a total volume of 100 μL. The maximum concentration of the peptide was 32 μM for both strains. The plate was incubated at 37 °C, 950 rpm for 24 h, and MIC values were determined. Starting from the 2nd day, an aliquot was taken from the well with the highest sub-inhibitory concentration of the peptide, diluted 1000 times with 2xMHB, and used as a starter culture for the next round of selection. Sequential passaging followed by MIC determination continued for 30 days. The control serial passages in the absence of the peptide were also performed. Bacteria capable of growing at the maximum possible concentration of acidocin A and control bacteria were passaged for three additional days on agar plates in the absence of the peptide (to confirm that the acquired resistance is stable), then the final MICs were determined again.

### 2.5. Membrane Permeability Assays

The ability of the peptides to disrupt the integrity of bacterial membranes was assessed using chromogenic substrates that are unable to enter the intact cell [26]. For these assays, *E. coli* strain ML-35p was chosen that constitutively expresses β-lactamase (secreted into the periplasmic space), β-galactosidase (that is active in cytoplasm), and lacks lactose permease. Nitrocefin, which is a substrate for lactamase, served as an indicator of increased outer membrane permeability. *o*-Nitrophenyl-β-D-galactopyranoside (ONPG), a substrate for β-lactamase, was used to assess the integrity of the cytoplasmic membrane. The bacterial cultures were grown to stationary phase in 3% TSB medium at 37 °C overnight. The cells were washed three times with cold 10 mM phosphate-buffered saline (PBS), pH 7.4 (173 mM NaCl, 2.7 mM KCl, 10 mM Na_2_HPO_4_, 1.76 mM KH_2_PO_4_), and diluted to the concentration of 3.3 × 10^7^ CFU/mL with 10 mM PBS (pH 7.4), containing increased concentration of NaCl (1.2%, or 205 mM) and dissolved nitrocefin (Merck-Millipore, Burlington, MA, USA) or ONPG (AppliChem GmbH, Darmstadt, Germany). Two-fold serial dilutions of the peptides were prepared in 50 µL of 0.1% BSA in a 96-well plate, and 150 µL of the cell suspension was added to each well. The final concentration of bacterial cells was 2.5 × 10^7^ CFU/mL, and the final concentrations of substrates were 2.5 mM ONPG or 20 µM nitrocefin in the final volume of 200 μL. The plate was incubated at 35 °C, 500 rpm, and the absorbance of the hydrolysis products of ONPG or nitrocefin was measured spectrophotometrically at regular intervals at 405 nm or 492 nm, respectively, using AF2200 microplate reader (Eppendorf, Hamburg, Germany). Control experiments were carried out under the same conditions with 0.1% BSA instead of peptides. For each peptide, three independent experiments were performed.

### 2.6. Hemolysis Assay

Hemolytic activity of acidocin A was tested against the fresh suspension of human red blood cells (hRBC) [27]. The experiments were conducted with the hRBC from blood samples of two independent donors (in triplicate for each). The hRBC were washed three times with PBS, pH 7.4, and resuspended in the same buffer at a concentration of 8% (*v*/*v*). Aliquots of 50 μL of hRBC suspension were added to 50 μL of two-fold serial dilutions of acidocin A, starting with 256 μM, in the wells of a round-bottom 96-well microplate to obtain a total volume of 100 μL, hRBC concentration of 4%, and a maximum peptide concentration of 128 μM. The suspension was incubated for 1.5 h at 37 °C in a plate shaker at 1000 rpm, then the plate was centrifuged at 3500 g for 10 min at 4 °C. Aliquots of 50 μL of the supernatants were transferred to a flat-bottom 96-well microplate, and the release of hemoglobin was monitored by measuring the absorbance at 405 nm in AF2200 microplate reader. hRBC in PBS and 0.1% Triton X-100 (Sigma-Aldrich, St. Louis, MO, USA) solution were used as negative and positive controls, respectively. The percentage of hemolysis was calculated according to the following equation: hemolysis (%) = [(A_S_ − A_0_)/(A_100_ − A_0_)] × 100%, where A_S_ is the absorbance of the sample, A_100_ is the absorbance of completely lysed hRBC in 0.1% Triton X-100, and A_0_ is the absorbance of base lysis in PBS.

### 2.7. Cytotoxicity Assay

The cytotoxic properties of acidocin A and avicin A were investigated by resazurin-based cell cytotoxicity assay [28,29]. The membranolytic peptide melittin from honeybee venom was used as a positive control. Primary peripheral blood mononuclear cells (PBMCs) collected from a healthy donor and human acute monocytic leukemia cell line (THP-1) were purchased from American Type Culture Collection (ATCC PCS-800-011 and ATCC TIB-202, respectively).

PBMCs and THP-1 cells were seeded into 96-well plates at 2 × 10^6^ and 1 × 10^6^ cells per well, respectively, in RPMI-1640 (Capricorn Scientific, Ebsdorfergrund, Germany), supplemented with 10% fetal bovine serum (FBS) (Capricorn Scientific, Ebsdorfergrund, Germany), and kept in CO_2-_incubator (5% CO_2_, 37 °C). After 24 h, the serial two-fold dilutions of the peptides in the culture medium were added to final concentrations from 0.25 to 64 μM. After 24 h of incubation with the peptides, resazurin was added at a final concentration of 70 µM, and the plates were incubated overnight (16 h) in CO_2_-incubator. Resorufin fluorescence was registered using 535/595 filter with AF2200 microplate reader. Untreated cells of both lines were used as negative controls. The cell viability was calculated as: cell viability (%) = (F_sample_/F_control_) × 100%. Cell lysis was observed with CKX41 microscope (Olympus, Shinjuku, Tokyo, Japan).

### 2.8. Measurement of Cytokine Response by Human Monocytes

Primary CD14^+^ blood monocytes were isolated from PBMCs by adherence according to standard technique [30], seeded into the wells of a 24-well plate filled with the complete RPMI-1640 medium supplemented with 10% human AB serum (HABS, Capricorn Scientific, Ebsdorfergrund, Germany) and 1X antibiotic-antimycotic solution (Invitrogen, Waltham, MA, USA) 24 h prior to the experiment, and kept in a humidified CO_2_-incubator (5% CO_2_, 37 °C). After 24 h, medium in each well was replaced by fresh complete RPMI-1640 medium with 10% HABS, supplemented with 2 µM of acidocin A or avicin A for the sample wells or fresh medium alone for the control wells. Cell cultures were kept in CO_2_-incubator (5% CO_2_, 37 °C) for another 24 h. Culture supernatants were collected 24 h later and stored at −70 °C degrees for less than one month prior to the analytes assessment.

The following 48 analytes were measured at a protein level by multiplex xMAP technology using the MILLIPLEX Human Cytokine/Chemokine/Growth Factor Panel A kit (HCYTA-60K-PX48) (Merck-Millipore, Burlington, MA, USA): sCD40L, EGF, Eotaxin-1/CCL11, FGF-2/FGF-basic, Flt-3 ligand, Fractalkine/CX3CL1, G-CSF, GM-CSF, GROα/CXCL1, IFNα2, IFNγ, IL-1α, IL-1β, IL-1RA, IL-2, IL-3, IL-4, IL-5, IL-6, IL-7, IL-8/CXCL8, IL-9, IL-10, IL-12(p40), IL-12(p70), IL-13, IL-15, IL-17A/CTLA8, IL-17E/IL-25, IL-17F, IL-18, IL-22, IL-27, IP-10/CXCL10, MCP-1/CCL2, MCP-3/CCL7, M-CSF, MDC/CCL22, MIG/CXCL9, MIP-1α/CCL3, MIP-1β/CCL4, PDGF-AA, PDGF-AB/BB, RANTES/CCL5, TGFα, TNFα, TNFβ, and VEGF-A. Multiplex-based assay read-out was performed using MAGPIX system with the xPONENT 4.2 software (Merck-Millipore, Burlington, MA, USA) in accordance with the manufacturer’s instruction with overnight incubation of the samples with primary antibodies. Final analysis was carried out with the MILLIPLEX Analyst v5.1 software (Merck-Millipore, Burlington, MA, USA). Release of the analytes in control and experimental samples was compared with unpaired two-sample *t*-test using GraphPad Prism v.8.0.1 (GraphPad Software, Inc, San Diego, CA, USA). The *p*-values < 0.05 were considered significant.

## 3. Results

### 3.1. Expression and Purification of the Recombinant Peptides

PLBs do not undergo post-translational modifications, except for the formation of disulfide bonds, so we used a well-established heterologous expression system based on *E. coli* BL21 (DE3) strain, which had previously been used to obtain avicin A [14]. In our experience, thioredoxin-PLB hybrid proteins are often characterized by low solubility and susceptibility to *C*-terminal proteolysis in *E. coli* cell lysates, so denaturing conditions were maintained during the ultrasonic processing and IMAC purification steps. The measured *m/z* values matched the corresponding calculated molecular masses (Figures S2 and S3, Table S1). The purity of the peptides, as assessed by Tris-tricin SDS-PAGE, was ≥ 95%; some of the samples contained traces of His8-thioredoxin and the non-cleaved fusion protein (Figure S4). The final yields were 2–8 mg per liter of culture (Table S1).

Acidocin A and avicin A preparations used in the cytokine response assay were repurified by RP-HPLC and were free of LPS, as assayed by LAL test. The assessed LPS level was below the detectable minimum of <0.0008 EU per mL in a final concentration of 2 µM for each peptide. The retention time of OR-7(VI) partially overlapped with the retention time of the thioredoxin carrier protein, so this peptide also required repeated purification.

Cys residues of the recombinant acidocin A were involved in the formation of disulfide bridge, as was evidenced by the alkylation experiment (Figure S5, Table S1).

### 3.2. Acidocin A Secondary Structure

**Figure 2:**
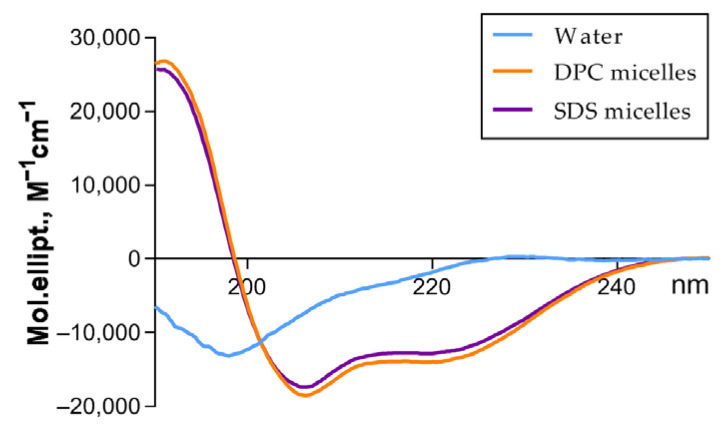
CD spectra for 200 µM acidocin A in aqueous solution and in the presence of DPC or SDS micelles.

The secondary structure of acidocin A was examined by CD spectroscopy (Figure 2, Table 1). The far-UV CD spectrum in water solution showed a combination of random coil and β-secondary structures. In the presence of the membrane-mimicking micelles of anionic (SDS) and zwitterionic (DPC) detergents, acidocin A increased the percentage of α-helices by reducing the proportion of disordered and β-sheet regions.

### 3.3. Antibacterial Activity

The MIC values of recombinant bacteriocins against a number of Gram-positive and Gram-negative bacteria are presented in Table 2 and Table 3, respectively. In contrast to the previously published data [11] for natural acidocin A, the recombinant peptide obtained in this work did not exhibit the activity against *L. monocytogenes*, even at the concentration of 128 μM. The peptide demonstrated a wide spectrum of antimicrobial activity against Gram-positive and Gram-negative bacteria of the genera *Bacillus*, *Mycobacterium*, *Lactococcus*, *Staphylococcus*, *Micrococcus*, *Acinetobacter*, *Pseudomonas*, and against strains of *E. coli*, including antibiotic-resistant ones. In contrast, avicin A was active only against *L. monocytogenes* EGD; its MICs against other strains were not determined as they were above 32 µM. Avicin A mutant with a Thr residue inserted into the pediocin box was totally inactive in the tested range of concentrations, while both variants of acidocin A, with and without the Thr residue, had identical antibacterial activity. OR-7(VI) variant was much less active than acidocin A against all tested bacterial strains. The MIC values of the AcdA(dT,C11S) and AcdA(dT,C31S) variants were, on average, 2–4 times higher compared to AcdA(dT) and acidocin A. The same decrease in antibacterial activity was observed for acidocin A and AcdA(dT) after reduction of the disulfide bond with DTT. The activity of the AcdA(dT,C11S,C31S) variant decreased significantly, especially against Gram-negative bacteria. The MIC values for acidocin A variants with mutations in the pediocin box or its deletion increased on average just by 2-fold and did not lead to a significant change in the antimicrobial activity of this peptide.

The activity of acidocin A and its mutants was found to be sensitive to salt (Table S3). The MIC against *E. coli* ML-35p in the presence of physiological NaCl concentration increased 8-fold (16 μM), whereas most other MICs exceeded the upper limit of the tested range of 32 μM.

Since the ability to prevent *Listeria* growth is an important marker of the specificity of PLB action, the presence of such activity in avicin A and its absence in the other tested peptides was qualitatively confirmed using the agar diffusion method (Figure S6).

None of the peptides tested in this work was active (MICs > 32 μM) against the following strains: *Staphylococcus aureus* ATCC 29213, *Enterococcus faecalis* ATCC 29212, *Bacillus megaterium* B-392, *Bacillus mycoides* B-414, *Proteus mirabilis* XDR CI 3423, *Klebsiella pneumonia* ATCC 700603.

### 3.4. Bacterial Resistance to Acidocin A

To investigate the mechanisms of acidocin A antibacterial activity and to identify its putative target we attempted to obtain resistant forms of two initially susceptible strains of bacteria: *E. coli* XDR CI 1057 (Gram-negative) and *B. licheniformi*s B-511 (Gram-positive). Previously, the first of them had demonstrated the ability to rapidly develop resistance to antimicrobial peptides of animal origin [27]. In our work, we used the method of sequential passaging of sensitive strains in continually increasing subinhibitory concentrations of antimicrobial agent. During the experiment, the MIC of acidocin A against *E. coli* XDR CI 1057 increased only 2-fold, and the MIC against *B. licheniformis* B-511—by a maximum of 8-fold, while the control cultures showed unchanged MICs. In both cases, after three days of passaging in the absence of the peptide, the MICs returned to their initial values. Since the phenotypic changes were not stable, they did not appear to be the result of mutations; for this reason, the genome sequencing of the obtained strains was not performed.

### 3.5. Permeability of E. coli ML35p Outer and Inner Membranes

Mechanism of action of many bacteriocins involves the formation of transmembrane pores that leads to disruption of membrane barrier function, dissipation of the transmembrane potential, and leakage of low molecular weight substances, including ATP, inorganic ions, amino acids, and other molecules [1]. The ability of acidocin A, avicin A, and their variants to disrupt the integrity of the cytoplasmic and outer membranes of *E. coli* ML-35p was studied spectrophotometrically using chromogenic substrates ONPG and nitrocefin. Melittin, which is a potent membranolytic, was chosen as a positive control.

**Figure 3:**
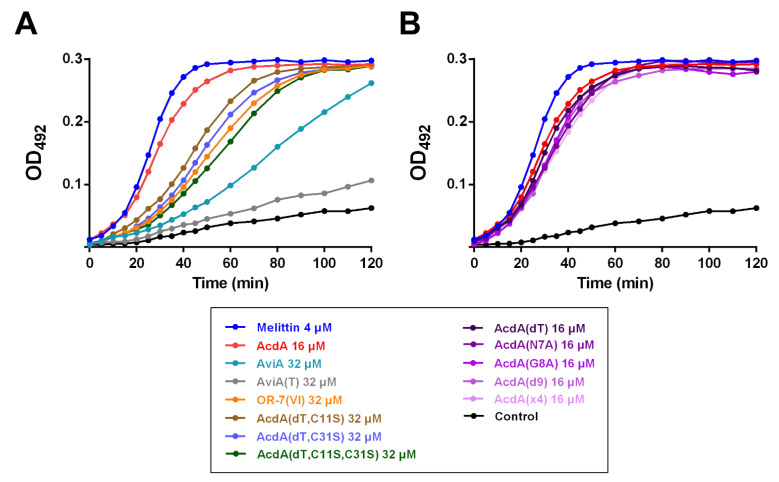
(**A**,**B**) Kinetic curves of *E. coli* ML-35p outer membrane permeability in the tests with nitrocefin. (**B**) Kinetic curves for acidocin A variants with mutations in the pediocin box that had a similar appearance shown separately and colored in purple with different intensities. Negative control demonstrates spontaneous hydrolysis of nitrocefin in the absence of peptides. The resulting curves are representative of three independent experiments; the shape of the curves was similar for each individual peptide.

The effects on the outer membrane of *E. coli* ML-35p are shown in Figure 3. All peptides, with the exception of AviA(T), were able to disrupt its integrity. The effect of acidocin A at the concentration equal to its MIC in the presence of 0.9% NaCl (Table S3) was comparable to the membranolytic effect of 4 μM melittin. All variants of acidocin A with mutations in the pediocin box showed similar kinetic curves to the original peptide. Compared to them, the membrane activity of acidocin A variants without the disulfide bond and OR-7(VI) was reduced. The effect of avicin A was even weaker.

**Figure 4:**
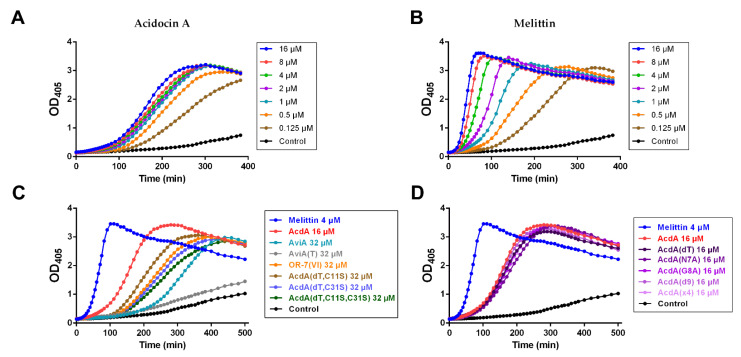
Kinetic curves of *E. coli* ML-35p cytoplasmic membrane permeability in the tests with ONPG. (**A**,**B**) Effects of acidocin A and melittin at different concentrations. (**C**,**D**) Comparative analysis of the effects demonstrated by different bacteriocins (the colors are identical to those used in Figure 3). Negative control demonstrates spontaneous hydrolysis of ONPG in the absence of the peptides. The resulting curves are representative of three independent experiments; the shape of the curves was similar for each individual peptide.

The effects on the cytoplasmic membrane of *E. coli* ML-35p are shown in Figure 4. In general, one could observe segregation into the same groups as in the previous experiment. Activity decreased in the following order: melittin >> acidocin A ≈ pediocin box mutants > peptides without a disulfide bond > avicin A >> AviA(T) (Figure 4C,D). There was more noticeable difference between melittin and acidocin A in this experiment (Figure 4A,B). In contrast to melittin, the concentration dependence of acidocin A membranolytic effect was negligible, starting from 1 μM and above.

As is often the case in studies involving various types of biological activity assays, we did not observe a simple correlation between the MIC values and the activity in these tests. For example, bacteriocin OR-7(VI), which was not active against most of the strains tested (including *E. coli* ML-35p), demonstrated results very close to AcdA(dT) C11S/C31S mutants. This particular case can be explained by the low proteolytic stability of bacteriocin OR-7(VI), which is more rapidly inactivated in the culture medium than in PBS solution.

### 3.6. Assessment of the Hemolytic Activity and Cytotoxicity on the Human Cell Lines

The hemolytic activity of acidocin A was tested on hRBC from two independent volunteers. At the maximum tested concentration of 128 μM, the peptide lysed only 3% of human erythrocytes; at lower concentrations, the percentage of hemolysis did not exceed statistical error. The cytotoxicity of acidocin A and avicin A against primary human PBMCs and THP-1 was evaluated by a resazurin reduction assay used as a method of indirect measurement of cell viability. In these experiments, acidocin A and melittin (control), but not avicin A, were toxic to both cell lines (Figure S7). PBMCs were more sensitive to the action of the peptides than the more rapidly dividing THP-1 cells. Melittin, as expected, showed pronounced toxic effects (cell viability less than 10%) on both types of human cells at concentrations of ≥16 μM. Microscopic analysis revealed the cell lysis in the presence of melittin in these concentrations (data not shown). Acidocin A demonstrated less cytotoxicity: the cell viability less than 10% was observed only at the concentration of 64 μM for both cell lines. The severity of the cytotoxic effect of acidocin A gradually declined as the time of cell incubation with resazurin increased from 2 to 24 h (not shown in the graph). Since acidocin A did not cause lysis of human cells, the toxic effects observed were probably associated with a decrease in metabolic activity and cell growth.

### 3.7. Immunomodulatory Effects on Human Immune Cells

**Figure 5:**
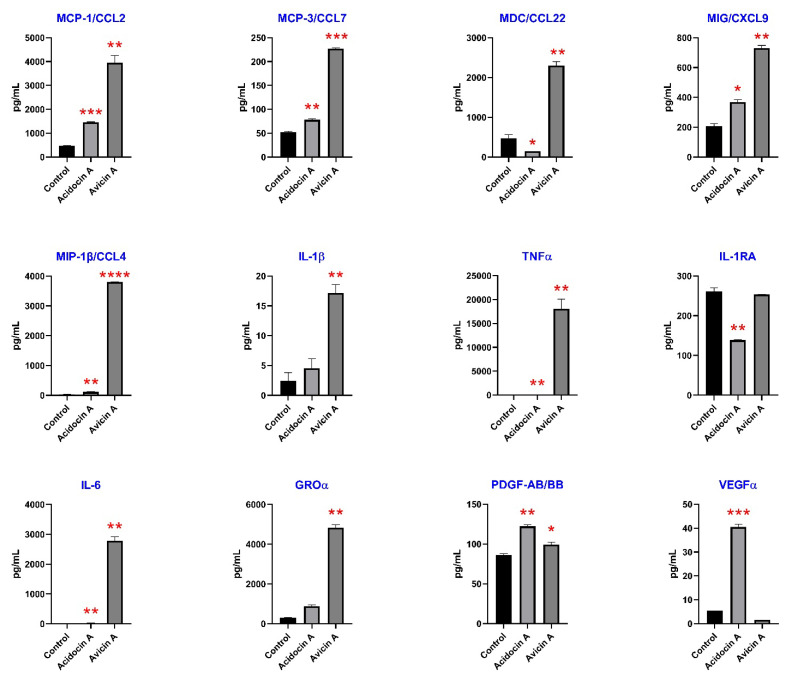
Profiles of cytokines, chemokines, and growth factors production in vitro by primary monocytes in response to incubation with 2 µM of bacteriocins. Error bars represent a standard deviation (±SD) between two replications. Significance levels are: * *p* < 0.05, ** *p* ≤ 0.01, *** *p* ≤ 0.001, **** *p* ≤ 0.001.

It was found that both bacteriocins increased the production of inflammatory chemokines MIG/CXCL9 (from 205 to 367 pg/mL, *p* < 0.05 for acidocin A, and to 729 pg/mL, *p* < 0.005 for avicin A), MCP-1/CCL2 (from 464 to 1450 pg/mL, *p* = 0.0005 for acidocin A, and to 3950 pg/mL, *p* < 0.005 for avicin A), MCP-3/CCL7 (from 52.14 to 78.11 pg/mL, *p* < 0.005 for acidocin A, and to 227 pg/mL, *p* = 0.0001 for avicin A), and MIP-1β (from 34.63 to 115.4 pg/mL, *p* < 0.005 for acidocin A, and to 3805 pg/mL, *p* < 0.0001 for avicin A) (Figure 5). Among them, monocyte chemoattractant protein-1 (MCP-1/CCL2) is one of the strongest known chemotactic factors for monocytes and a key chemokine that regulates migration and infiltration of monocytes/macrophages [31]. Interestingly, acidocin A inhibited the production of anti-inflammatory macrophage-derived chemokine (MDC/CCL22) from 463 to 142 pg/mL, *p* < 0.05, but avicin A increased its production to 2303 pg/mL, *p* < 0.005. MDC is a potent attractant for CCR4 expressing polarized Th2 and Tc2 cells [32]. Both bacteriocins also increased inflammatory cytokines IL-6 (from 4.48 to 26.69 pg/mL, *p* < 0.005 for acidocin A, and to 2787 pg/mL, *p* < 0.005 for avicin A) and TNFα (from 67 to 132 pg/mL, *p* = 0.001 for acidocin A, and to 17,982 pg/mL, *p* < 0.01 for avicin A) (Table S4). Only acidocin A inhibited the production of anti-inflammatory IL-1RA (from 261 to 138 pg/mL, *p* < 0.005), and only avicin A increased the production of proinflammatory IL-1β from 2.28 to 17.13 pg/mL, *p* < 0.01 by primary monocytes.

It was also shown that acidocin A increased the production of growth factors PDGF-AB/BB (from 86 to 122 pg/mL, *p* < 0.005) and VEGFα (from 5.44 to 40.51 pg/mL, *p* < 0.001) while avicin A induced slight elevation of only PDGF-AB/BB from 86 to 99 pg/mL, *p* < 0.05. Both bacteriocins increased the production of GROα/CXCL1 (from 311 to 876 pg/mL, *p* < 0.01 for acidocin A, and to 4832 pg/mL, *p* < 0.001 for avicin A), which exhibits dual role as an attractant for neutrophils similar to interleukin-8 (IL-8/CXCL8) and can play a role in angiogenesis and arteriogenesis [33]. Avicin A induced the production of G-CSF, which stimulates proliferation of neutrophils, from below the detectable minimum of <3.18 pg/mL to 190 pg/mL.

## 4. Discussion

In the original publication by Kanatani et al. (1995), the antagonistic activity of *L. acidophilus* TK9201 and the inhibitory spectrum of purified acidocin A were determined using tests on a solid medium [11]. In our work, we used the method of serial dilutions in a liquid medium, which, in contrast to the above, allowed us to determine the MIC values of the tested compounds. Our data differ significantly from the results published earlier: although we managed to reproduce the activity against *L. lactis*, the recombinant acidocin A did not show activity against *L. monocytogenes* and, unlike the natural peptide, inhibited the growth of *Bacilli* and several strains of *E. coli* (Table 2 and Table 3). Given the identity of *m/z* values, these discrepancies could be explained by differences in the folding of the peptides in different preparations. Unfortunately, there is no information in the literature about the spatial structure of natural acidocin A. Our studies showed that the recombinant peptide contained an intramolecular disulfide bond (Figure S5) and, according to CD spectroscopy, had a high percentage of ordered regions at the level of the secondary structure (Table 1). No satellite peaks with the same *m/z*, which could indicate the presence of alternatively folded forms, were observed in the HPLC chromatograms (Figure S1). The CD spectra of recombinant acidocin A resembled the spectra of previously obtained recombinant avicin A, which exhibited highly specific activity against *L. monocytogenes* in the nanomolar concentration range and was considered to be completely identical to the natural peptide [14]. Both peptides formed α-helices in the membrane-mimicking environment of micelles of zwitterionic and anionic detergents. In acidocin A, the increase in the proportion of α-helical fold was more pronounced. Unlike avicin A, it was accompanied by a major decrease in the contribution of β-folded regions. The pattern of conformational changes in both peptides was almost independent of the type of detergent.

According to the published data, folding of PLBs containing a single disulfide bond does not require the participation of auxiliary proteins and usually does not cause problems in heterologous expression experiments [34]. Along with the results of structural studies, this led us to the idea that the difference in the activity spectra of recombinant and natural acidocin A is due to the choice of different indicator strains and testing techniques. We repeated antimicrobial tests on *Listeria* using the agar diffusion method but obtained the same negative results for acidocin A (Figure S6). Avicin A, again, showed high activity typical of PLBs. It cannot be ruled out, though, that the authors of the original publication, presenting data on the “inhibitory spectrum of bacteriocin”, could have in mind the antagonistic activity of the producer strain—in our opinion, the reference to the testing method they cited [24] leaves the possibility of such an interpretation. In this case, the data they published referred to the total antimicrobial activity of *L. acidophilus* TK9201 culture, which, along with acidocin A, might contain other antimicrobial substances.

A broad spectrum of the acidocin A activity was observed in our experiments, including that against Gram-positive and Gram-negative bacteria. This fact, together with rather high (micromolar) MIC values, distinguished it from avicin A, the representative of classical PLBs, which target a limited set of bacterial strains in the nanomolar concentration range. For many AMPs, including most PLBs and lipid II-targeting lantibiotics, the outer membrane of Gram-negative bacteria is a formidable barrier that prevents the manifestation of antimicrobial activity. Permeability assays using chromogenic substrates on *E. coli* ML-35p confirmed the ability of acidocin A to damage both outer and inner membranes, which brings it closer to such nonselective membranolytic peptides as melittin from honeybee venom (Figure 3 and Figure 4).

The only characterized homologue of acidocin A, bacteriocin OR-7, is known to be active against Gram-negative bacteria of the species *Campylobacter jejuni* [12]. This peptide also contains an additional Thr residue in the pediocin box, but differs from acidocin A in the central part of amino acid sequence and contains only one Cys residue. The presence of two Met residues did not allow the use of cyanogen bromide cleavage in the purification procedure. OR-7-containing fusion proteins with different carriers were characterized by either low solubility or low proteolytic stability, which became an obstacle to the use of enzymatic hydrolysis as an alternative to chemical digestion. Due to these reasons, we decided to obtain a mutant variant, named OR-7(VI), with the replacement of Met residues by Val and Ile residues, which have close hydrophobicity and size and are found in similar positions in the acidocin A sequence (Figure 1A,B). MALDI MS analysis showed that the only available Cys residue was not involved in the formation of an intermolecular disulfide bond, and the peptide existed in a monomeric form. In contrast to acidocin A, the addition of DTT to OR-7(VI) samples prior to antimicrobial testing did not affect the activity. Overall, MIC values for OR-7(VI) were significantly higher than those obtained for acidocin A and, contrary to expectations, there was no activity on Gram-negative bacteria (Table 2 and Table 3). Although the antimicrobial activity of OR-7(VI) was markedly lower than that of AcdA(dT) C11S/C31S mutants, the results obtained for all the peptides lacking the disulfide bond were very close in membrane permeability tests (Figure 3A and Figure 4C). The relatively lower antimicrobial activity of OR-7(VI) can be explained by the lower net positive charge of the molecule compared to acidocin A (roughly, +6 vs. +10 at pH 7.0). According to the generally accepted model, cationic AMPs are attracted to the negatively charged surface of the bacterial membrane or cell wall components [1,35,36]. Increasing the ionic strength of the solution almost always reduces their antimicrobial activity [27,37,38]. The increase in MICs of acidocin A in the presence of physiological NaCl concentration (Table S3) highlights the importance of electrostatic interactions in the mechanism of its antimicrobial action. However, as in many such cases, the fundamental question of how a membrane-targeting AMP exerts its effects in biological media containing large amounts of ions and biopolymers carrying charged chemical groups remains open.

At present, it is known that the IIC and IID subunits of Man-PTS in the cytoplasmic membrane of bacteria form the binding site for PLBs, and the activity spectrum is determined by the phylogenetic distribution of those Man-PTS variants, for which the peptide has the highest affinity [39,40]. Recently, cryo-electron microscopy structures of Man-PTS from *L. monocytogenes* alone and its complex with pediocin PA-1 [7] and bacteriocin-receptor-immunity ternary complex from *Latilactobacillus sakei* (previously assigned to the genus *Lactobacillus*) [41] were reported, which provided the solid basis for pore formation and immunity mechanism models. Sequential passaging of sensitive strains in continually increasing subinhibitory concentrations of receptor-targeting AMPs usually allows for selection of resistant forms of bacteria whose genome analysis shows the emergence of mutations in certain regions of the receptor genes [42,43]. However, our attempts to obtain acidocin A-resistant strains of *B. licheniformis* and *E. coli* were unsuccessful. It can be assumed that the mechanism of action of this bacteriocin against the strains used in our experiment does not involve specific binding to the protein target, so the formation of resistance phenotype requires more significant shifts in the membrane chemical composition and surface charge, similar to those observed in experiments with low-selective membranolytic peptides [44]. AMPs with such a mechanism of action are usually more tolerant to changes in their structure. Indeed, amino acid substitutions in the pediocin box, or even deletion of it, detrimental to classical PLBs [45,46], resulted in only a moderate decrease in the antimicrobial (Table 2 and Table 3) and membrane permeabilization (Figure 3B and Figure 4D) activities of acidocin A. At the same time, the insertion of an additional Thr residue into the pediocin box of avicin A led to a complete loss of activity against *L. monocytogenes* (Table 2 and Table 3, Figure S6). It remains unclear why the deletion of the “noncanonical” Thr residue in acidocin A, contrary to other mutations, had almost no effect on the MIC values and also why the insertion of Thr resulted in the decrease in avicin A activity against *E. coli* ML-35p membranes, taking into account that the strain is not susceptible to the wild-type peptide. We also were unable to explain the sharp decrease in activity against Gram-negative bacteria observed for Acd(dT) C11S + C31S double mutant.

Due to the narrowly targeted mode of action, bacteriocins usually do not have a pronounced toxic effect on various human cells at concentrations significantly exceeding their MICs [47]. For example, such bacteriocins as nisin, pediocin PA-1, and bactofencin A show low cytotoxicity against epithelial cells Caco-2 in experiments in vitro [48]. Penisin does not exhibit cytotoxicity towards HeLa (human cervical cancer cells), RWPE1 (human prostate epithelial cells), and RAW cells (mouse macrophages) macrophage cell lines [49]. However, there are known exceptions. Microcin J25 is not toxic to erythrocytes and murine macrophage RAW264.7 cells [50], but shows toxicity towards rat heart mitochondria at low concentrations [51]. Cinnamycin displays high toxicity against both erythrocytes and HeLa cells due to its ability to bind phosphatidylethanolamine of the plasma membranes [52]. Our results obtained on bacterial models suggested that acidocin A might be another exception to this rule. However, in tests of hemolytic activity, it did not show much cell lysis at concentrations of up to 128 μM. Unlike avicin A, it reduced the viability of human immune cells, PBMCs and THP-1. However, its toxic effects were manifested in concentrations significantly higher than the MICs obtained for most of the tested bacterial strains. The cytotoxic effect of acidocin A was not lytic, but apparently was associated with a decrease in the metabolic activity of the cells.

The low toxicity of acidocin A and avicin A allowed us to explore their immunomodulatory potential. This aspect is rarely addressed in bacteriocin studies [47]. It has been previously shown that bacteriocin BacSp222 from commensal bacterium *Staphylococcus pseudintermedius* also exhibits proinflammatory properties due to stimulation of the production of proinflammatory TNFα, MCP-1, and IL-1α by murine macrophage-like cell line RAW 264.7 [53]. Lantibiotic nisin Z also induced the secretion of MCP-1 chemokine together with neutrophil attractants IL-8/CXCL8 and GROα [54]. Nisin A has been shown to have proliferative activity on porcine peripheral blood leucocytes, it stimulated pro-inflammatory IL-1β and IL-6 cytokines production, and increased the percentage of double positive CD4^+^CD8^+^ T cells among unstimulated leucocytes [55]. For this work, we used primary human monocytes, as monocytes and macrophages are critical for innate immunity. Homeostatic control of monocytes/macrophages is shared between pro- and anti-inflammatory molecules and is essential for the regulation of the immune system [56]. We showed that both peptides exhibit proinflammatory properties, increasing the production of proinflammatory cytokines and chemokines (MIG, MCP-1, MCP-3, MIP-1α, MIP-1β, IL-6, and TNFα); however, avicin A activity was much higher (Figure 5). The immunomodulatory properties of LAB bacteriocins are important, not only in terms of their potential use as drugs, but also in the aspect of host–symbiont relationships. The next step in this direction could be to evaluate the ability of these compounds to cross the intestinal epithelial cells layer and the enterohematic barrier, which would allow them to modulate the host immunity at the local and systemic level.

Among the many biological effects exhibited by animal and human host-defense peptides, the ability to accelerate wound healing is sometimes observed. Similar activity has been detected in some bacteriocins. For instance, nisin A has been shown to be a potential treatment to use in wound healing, as it increases the mobility of skin cells, dampens the effect of lipopolysaccharide and proinflammatory cytokines, and decreases bacterial growth [57]. Growth factors such as platelet-derived growth factor (PDGF), fibroblast growth factor (FGF), and epidermal growth factor (EGF) play an important role in the first phase of wound healing—fibrin clot formation [58]. We observed a moderate, but statistically significant, increasing of PDGF by primary monocytes in response to incubation with both bacteriocins, especially with acidocin A. This peptide also increased the production of FGF-2 (Table S4) and vascular endothelial growth factor (VEGF) (Figure 5). VEGF stimulates wound healing via multiple mechanisms, including collagen deposition, angiogenesis, and epithelization [59]. The next phase after fibrin clot formation is inflammation, a normal part of the wound-healing process, which is important for the removal of contaminating microorganisms [58]. This phase is sustained by TNFα, IL-1 and pro-inflammatory chemokines, many of which are induced in response to incubation of monocytes with both bacteriocins. Thus, the cytokine induction profiles suggest that bacteriocins studied in this work might also have wound healing properties—however, this speculation should be validated.

## 5. Conclusions

According to our data, acidocin A differs markedly from typical PLBs, not only in its amino acid sequence, but also in the spectrum of activity and mode of action on bacterial membranes. Its tolerance to mutations in the pediocin box raises questions about the functional significance of this conservative amino acid block for this peptide. It is possible that acidocin A still exhibits a specific receptor-mediated activity against some groups of bacteria that remained outside the scope of our work. The described immunomodulatory effects of acidocin A and avicin A are of particular interest due to the scarcity of information on this topic in the literature on PLBs, despite the wide distribution of the producers of these compounds in human and animal microbiota. We showed that both peptides exerted pro-inflammatory effects on primary human monocytes and increased expression levels of several growth factors crucial for wound healing. The results obtained for avicin A likely can be extrapolated to its closest homologues. Although PLBs are known for their rather low proteolytic stability, it is worth investigating the possible effects they may exhibit immediately after secretion by symbiotic or probiotic bacteria into the lumen of the human intestinal tract.

## Figures and Tables

**Table 1:** Acidocin A and avicin A secondary structure estimation (%) predicted from far-UV CD spectra.

Bacteriocin	Condition	α-Helix, %	β-Sheet, %	β-Turn, %	Random, %	NRMSD
Acidocin A	Aqueous solution	5.7	33.7	22.6	37.9	0.02
	DPC micelles	44.0	8.1	19.2	28.6	0.01
	SDS micelles	40.2	10.6	20.3	28.8	0.01
Avicin A *	Aqueous solution	5.8	32.8	22.1	39.3	0.02
	DPC micelles	19.4	28.6	21.6	27.8	0.02
	SDS micelles	19.7	29.3	23.1	30.4	0.02

* These values were calculated from the previously published CD spectra of recombinant avicin A [14].

**Table 2:** Antibacterial activity of bacteriocins against Gram-positive bacteria.

Bacteriocins	Minimum Inhibitory Concentration (µM) *							
	*L. monocytogenes* EGD	*L. lactis* MK66	*L. lactis* MK43	*B. subtilis* B-886	*B. licheniformis* B-511	*M. phlei* Ac-1221	*M. luteus* Ac-2229	*S. aureus* 209P
Acidocin A	>128	0.5	1	2	2	2	8	8
AcdA(dT)	>128	0.5	1	2	2	2	8	8
AcdA (DTT reduced)	>32	1	2	4	4	4	16	16
AcdA(dT) (DTT reduced)	>32	1	2	4	4	4	16	16
AcdA(dT,C11S)	>32	1	4	4	4	4	16	32
AcdA(dT,C31S)	>32	1	2	4	4	4	16	32
AcdA(dT,C11S,C31S)	>32	1	4	4	4	8	32	>32
AcdA(N7A)	>32	2	8	8	4	2	16	8
AcdA(G8A)	>32	2	4	8	8	2	16	8
AcdA(d9)	>32	2	8	8	8	4	16	16
AcdA(x4)	>32	2	4	8	4	4	8	16
OR-7(VI)	>64	4	>32	16	8	32	>32	>32
OR-7(VI) (DTT reduced)	>32	4	>32	16	8	32	>32	>32
Avicin A **	<0.125	>32	>32	>32	>32	>32	>32	>32
AviA(T)	>32	>32	>32	>32	>32	>32	>32	>32

* Green color indicates high antimicrobial activity; yellow and orange colors show reduced antimicrobial activity. Low antimicrobial activity is shown in light red and its absence—in bright red. ** The MIC of avicin A was outside the selected range of tested concentrations [14].

**Table 3:** Antibacterial activity of bacteriocins against Gram-negative bacteria.

Bacteriocins	Minimum Inhibitory Concentration (µM) *					
	*E. coli* ML-35p	*E. coli* ATCC 25922	*E. coli* SQ110	*E. coli* XDR CI 1057	*P. aeruginosa* MDR CI 1995	*A. baumannii* XDR CI 2675
AcdA	2	4	2	8	16	4
AcdA(dT)	2	4	1	8	16	4
AcdA (DTT reduced)	4	8	4	16	>32	8
AcdA(dT) (DTT reduced)	4	8	4	16	>32	8
AcdA(dT,C11S)	4	8	4	16	>32	8
AcdA(dT,C31S)	8	8	8	16	>32	16
AcdA(dT,C11S,C31S)	>64	>64	16	>64	>32	>32
AcdA(N7A)	8	16	8	8	16	4
AcdA(G8A)	8	16	4	8	16	8
AcdA(d9)	4	16	8	8	16	4
AcdA(x4)	8	16	8	8	16	4
OR-7(VI)	>32	>32	>64	>64	>32	>32
OR-7(VI) (DTT reduced)	>32	>32	>32	>32	>32	>32
AviA	>32	>32	>32	>32	>32	>32
AviA(T)	>32	>32	>32	>32	>32	>32

* Green color indicates high antimicrobial activity; yellow and orange show reduced antimicrobial activity. Low antimicrobial activity is shown in light red and its absence—in bright red.

## Data Availability

All data presented in this study are available from the corresponding author on reasonable request.

## Supplementary Materials

The following supporting information can be downloaded at: https://www.mdpi.com/article/10.3390/membranes12121253/s1, Figure S1: (A) Reversed-phase high-performance liquid chromatography (RP-HPLC) of the recombinant acidocin A; (B) Repurification of acidocin A sample by RP-HPLC; Figure S2: MALDI-TOF mass-spectrum of the recombinant acidocin A; Figure S3: MALDI-TOF mass-spectra of recombinant acidocin A variants, OR-7(VI), avicin A (AviA) and its mutant variant Avi(T); Figure S4: Tris-tricine SDS-PAGE analysis of recombinant bacteriocins; Figure S5: MALDI-TOF mass-spectra of the recombinant acidocin A (AcdA) after incubation with iodoacetamide (IAA) without (A) or after (B) the addition of 1,4-dithiothreitol (DTT); Figure S6: Agar diffusion assay of bacteriocins antimicrobial activity against *L. monocytogenes* EGD; Figure S7: Cytotoxic effects of different concentrations of acidocin A on PBMCs (A) and THP-1 cells (B) after 24 h of incubation; Table S1: Characteristics of recombinant bacteriocins obtained in this work; Table S2: Bacterial strains used in this study; Table S3: Antimicrobial activity of bacteriocins against some Gram-positive and Gram-negative bacteria in the presence of NaCl. Reference [60] is cited in the supplementary materials. Table S4: Absolute levels of 48 cytokines, chemokines and growth factors assessed by multiplex xMAP technology. Values highlighted in black indicate the values fall below the low detectable limit, in red-above the detectable maximun, while in blue indicate the data are in the detectable range.
