# Supplementary material for: Antimicrobial Activity and Immunomodulatory Properties of Acidocin A, the Pediocin-like Bacteriocin with the Non-Canonical Structure
Source: Membranes (Basel). 2022 Dec 11;12(12):1253. doi: 10.3390/membranes12121253 (PMC9780942; doi:10.3390/membranes12121253)
Supplement: Supplementary file 1 [file membranes-12-01253-s001.zip › membranes-2102677-supplementary-Figures S1-S7, Tables S1-S3.pdf]

Supplementary information for

Antimicrobial Activity and Immunomodulatory Properties of Acidocin A, the Pediocin-like Bacteriocin with the Non-Canonical Structure

Daria V. Antoshina [1,†], Sergey V. Balandin [1,*,†], Ivan V. Bogdanov [1], Maria A. Vershinina [1], Elvira V. Sheremeteva [1], Ilia Yu. Toropygin [2], Ekaterina I. Finkina [1], and Tatiana V. Ovchinnikova [1,3,4]

[1] M.M. Shemyakin and Yu.A. Ovchinnikov Institute of Bioorganic Chemistry, Russian Academy of Sciences, 117997 Moscow, Russia
[2] V.N. Orekhovich Research Institute of Biomedical Chemistry, 119121 Moscow, Russia
[3] Moscow Institute of Physics and Technology, 141700 Dolgoprudny, Moscow region, Russia
[4] Department of Bioorganic Chemistry, Faculty of Biology, Lomonosov Moscow State University, 119234 Moscow, Russia
* Correspondence: arenicin@mail.ru
† These authors contributed equally to this work.

Table S1. Characteristics of recombinant bacteriocins obtained in this work

Peptide	Final yield of recombinant peptide, mg/L	Hydrophobicity index *	Retention time during RP-HPLC, min	Calculated $[M+H]^+$ monoisotopic mass, Da *	Experimental $[M+H]^+$ monoisotopic mass, Da
Acidocin A	6.2	−0.298	47.2	6500.6	6500.2
AcdA(dT)	7.5	−0.291	46.8	6397.5	6396.8
AcdA(dT,C11S)	2.3	−0.349	47.9	6382.2	6382.5
AcdA(dT,C31S)	4.6	−0.349	48.1	6382.6	6382.4
AcdA(dT,C11S,C31S)	3.7	−0.407	48.5	6367.5	6367.1
AcdA(N7A)	7.1	−0.207	43.2	6459.7	6459.3
AcdA(G8A)	5.2	−0.260	44.6	6512.6	6513.2
AcdA(d9)	6.8	−0.190	46.3	5515.1	5514.1
AcdA(x4)	6.3	−0.298	45.4	6498.6	6498.8
OR-7(VI)	4.4	−0.556	49.5	6161.2	6161.7
Avicin A	5.4	−0.188	35.8	4288.7	4289.3
AviA(T)	4.9	−0.200	33.2	4389.1	4389.5
Acidocin A + IAA + DTT	-	-	-	6616.6	6616.5

* Expasy ProtParam tool was used to calculate the Kyte-Doolittle grand average hydropathicity indexes (GRAVY) and $[M+H]^+$ monoisotopic masses of recombinant peptides. The maximum and minimum values of hydrophobicity index are +4.5 for Ile and −4.5 for Arg. $[M+H]^+$ monoisotopic masses were calculated taking into account the presence of two Cys residues in acidocin A, AcdA(dT), AcdA(N7A), AcdA(G8A), AcdA(d9), AcdA(x4), avicin A, AviA(T) which form two disulfide bonds. $[M+H]^+$ monoisotopic mass for acidocin A after incubation with iodoacetamide (IAA) and 1,4-dithiothreitol (DTT) were calculeted taking into account the addition of two acetamide groups to Cys residues. Experimental $[M+H]^+$ monoisotopic masses were determined using MALDI-TOF mass spectrometry.

Table S2. Bacterial strains used in this study.

Bacterial strain	Characteristics (source, antibiotic resistance, etc.)
**Gram-positive bacteria**	
Bacillus licheniformis B-511	Laboratory strain (VKM collection)
Bacillus megaterium B-392	Laboratory strain (VKM collection)
Bacillus mycoides B-414	Laboratory strain (VKM collection)
Bacillus subtilis B-886	Laboratory strain (VKM collection)
Enterococcus faecalis ATCC 29212	Laboratory strain (ATCC collection)
Lactococcus lactis ssp. *lactis* MK43	Laboratory strain (collection of SEC IBCH RAS)
Lactococcus lactis ssp. *lactis* bv. *diacetylactis* MK66	Laboratory strain (collection of SEC IBCH RAS)
Listeria monocytogenes EGD	Laboratory strain (collection of FSBSI "IEM")
Micrococcus luteus Ac-2229	Laboratory strain (VKM collection)
Mycobacterium phlei Ac-1291	Laboratory strain (VKM collection)
Staphylococcus aureus ATCC 29213	Laboratory strain (ATCC collection)
Staphylococcus aureus 209P	Laboratory strain (ATCC collection)
**Gram-negative bacteria**	
Acinetobacter baumannii (XDR CI 2675)	Extensively drug resistant clinical isolate* (MBL+)
Escherichia coli ML-35p	Laboratory strain (ATCC collection)
Escherichia coli SQ110	Laboratory strain (provided by prof. A.S. Mankin [1])
Escherichia coli (XDR CI 1057)	Extensively drug resistant clinical isolate (urine, urinary tract infection; ESBL+)
Escherichia coli ATCC 25922	Laboratory strain (ATCC collection)
Klebsiella pneumonia ATCC 700603	Laboratory strain (ATCC collection)
Proteus mirabilis (XDR CI 3423)	Extensively drug resistant clinical isolate* (MBL+)
Pseudomonas aeruginosa (MDR CI 1995)	Multidrug resistant clinical isolate*

CI, clinical isolate; *, no data available on strain source; MDR, multidrug-resistant strain; XDR, extensively drug resistant strain; ESBL+, extended spectrum beta-lactamase producing strain; MBL+, metallo-beta-lactamase producing strain.

Table S3. Antimicrobial activity of bacteriocins against some Gram-positive and Gram-negative bacteria in the presence of NaCl

Bacteriocins	Minimum inhibitory concentration (μM) *					
	B. subtilis B-886	*B. licheniformis* B-511	*L. lactis* MK66	*L. lactis* MK43	*E. coli* ML-35p	*E. coli* SQ110
**Acidocin A**	>32	>32	32	>32	16	8
**AcdA(dT)**	>32	>32	32	>32	16	8
**AcdA(dT,C11S)**	>32	>32	>32	>32	32	16
**AcdA(dT,C31S)**	>32	>32	>32	>32	32	16
**AcdA(dT,C11S,C31S)**	>32	>32	>32	>32	n.t.	>32
**AcdA(N7A)**	>32	>32	>32	>32	16	32
**AcdA(G8A)**	>32	>32	>32	>32	16	32
**AcdA(d9)**	>32	>32	>32	>32	16	32
**AcdA(x4)**	>32	>32	>32	>32	16	32
**OR-7(VI)**	>32	>32	>32	n.t.	n.t.	n.t.

* To study the effect of the presence of inorganic salts on the activity of bacteriocins, MIC values were determined in Mueller-Hinton broth (MHB) supplemented with 0.9% NaCl (physiological concentration). *Light-orange* and *orange* color indicate reduced antimicrobial activity, while *light-red* and *red* color indicate very low antimicrobial activity or its absence, respectively; n.t.—not tested due to lack of activity in MHB in the absence of salt.

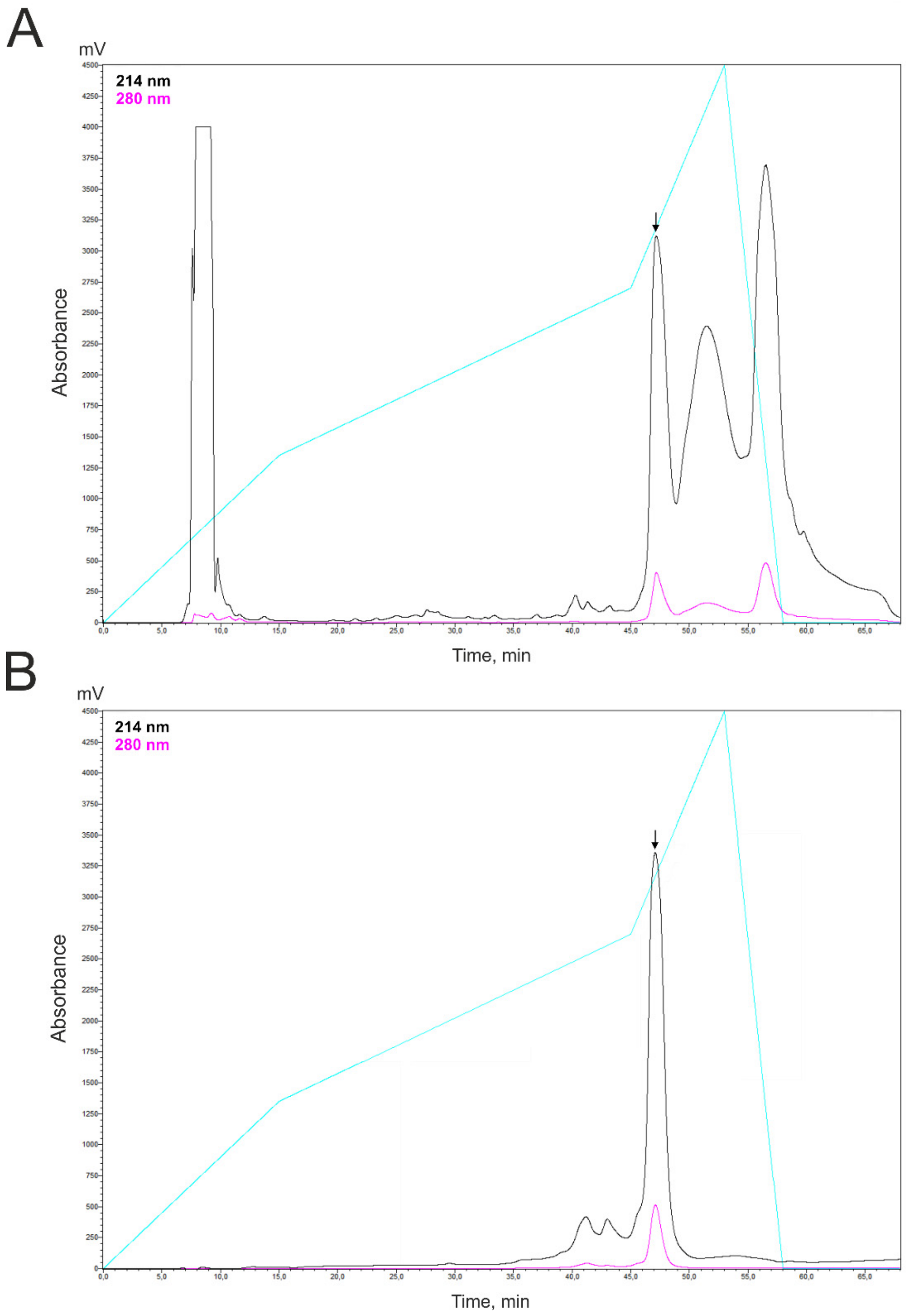

Figure S1. (A) Reversed-phase high-performance liquid chromatography (RP-HPLC) of the recombinant acidocin A. RP-HPLC was performed with a gradient (*bright blue* line) from 5 to 80% (v/v) of acetonitrile in water containing 0.1% TFA for 58 min. Peptide elution was detected spectrophotometrically by increasing absorbance at 214 and 280 nm (*black* and *pink* lines). The collected recombinant peptide fraction is marked with an arrow. Chromatograms of acidocin A mutants had a similar shape and close retention times. **(B)** Repurification of acidocin A sample by RP-HPLC.

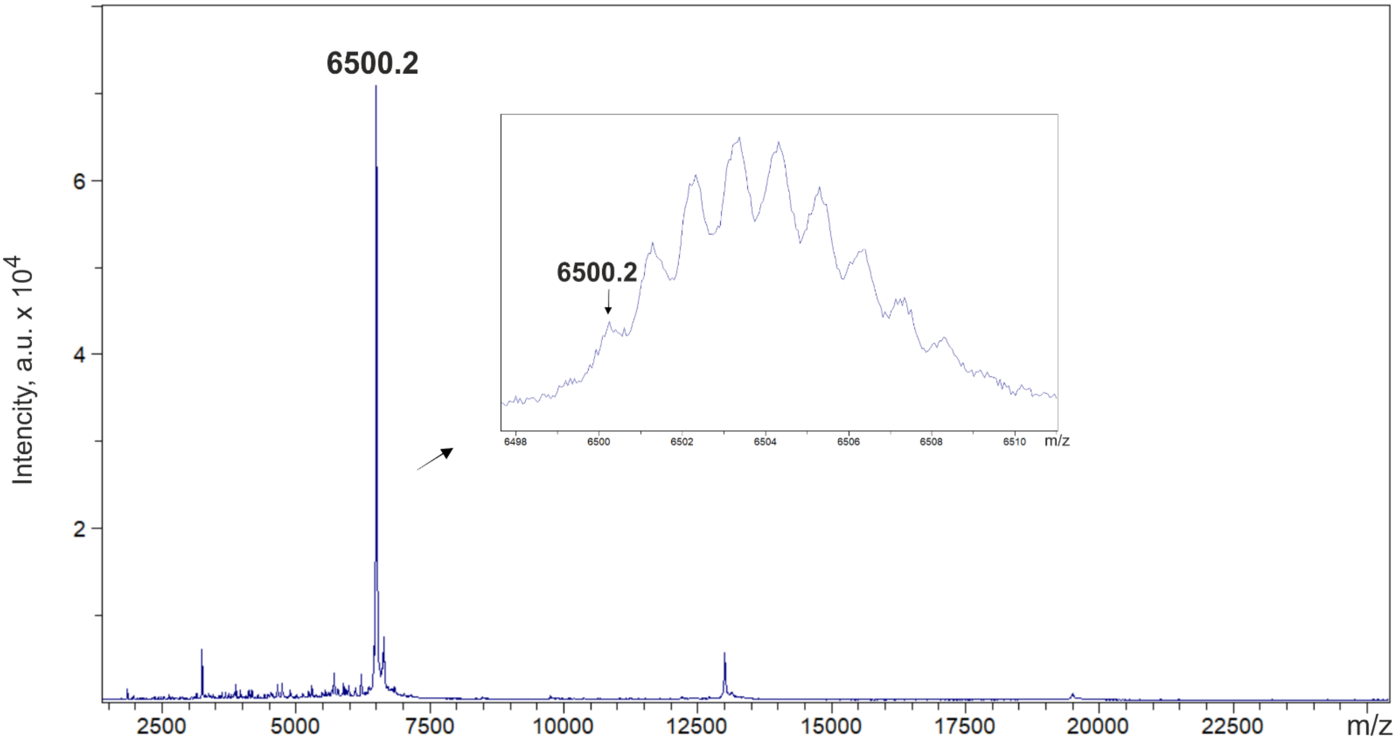

Figure S2. MALDI-TOF mass-spectrum of the recombinant acidocin A. The calculated *m/z* $[M+H]^+$ value for acidocin A containing one disulfide bond is 6500.6.

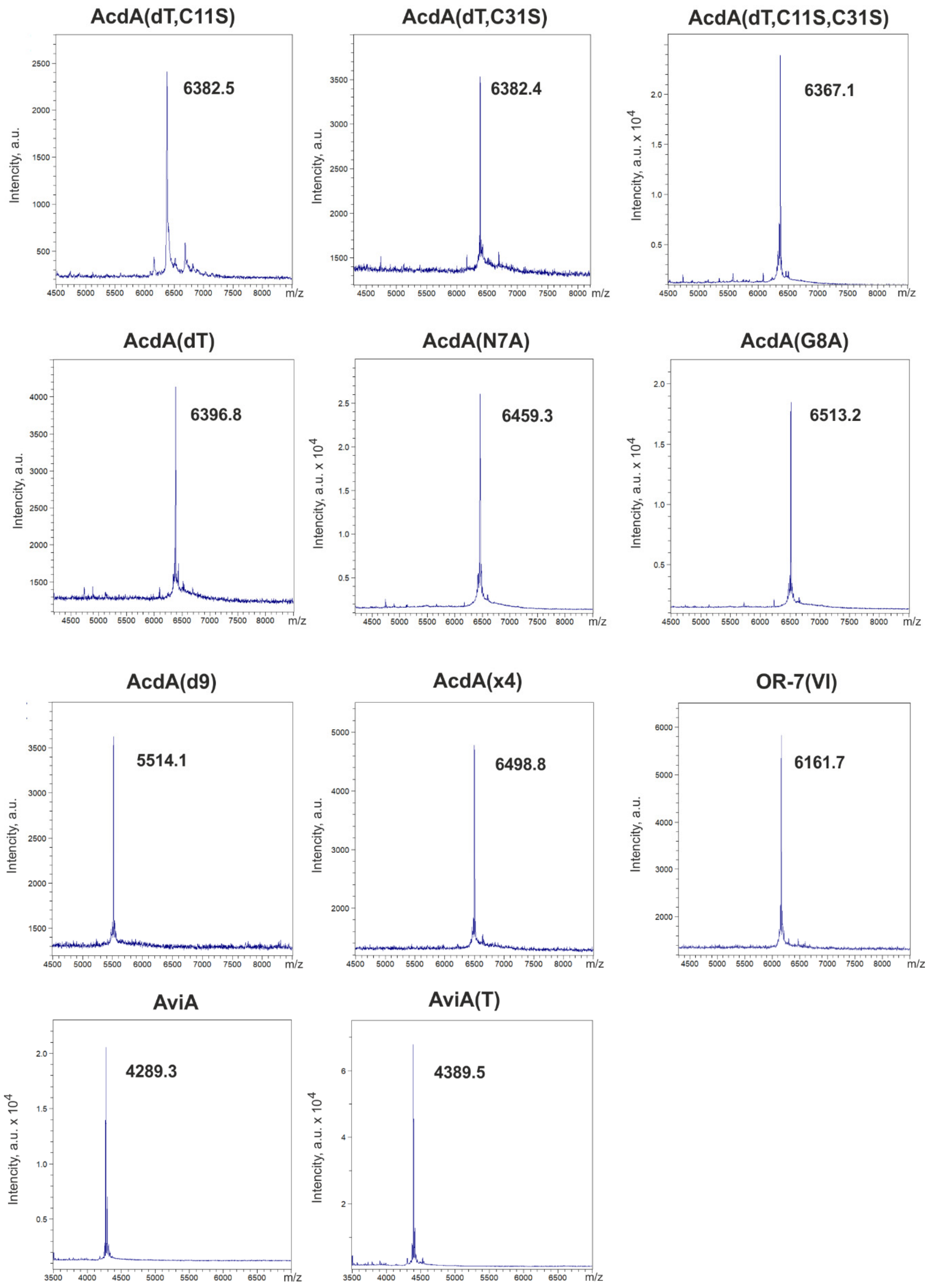

Figure S3. MALDI-TOF mass-spectra of recombinant acidocin A variants, OR-7(VI), avicin A (AviA) and its mutant variant Avi(T). The calculated *m*/*z* [M+H]$^+$ values for these peptides are given in the Table S1.

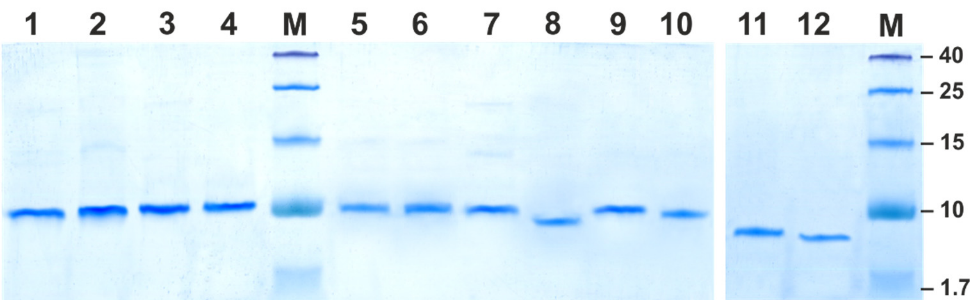

Figure S4. Tris-tricine SDS-PAGE analysis of recombinant bacteriocins. M—molecular weight marker with a range of molecular weights from 1.7 to 40 kDa. **1**—acidocin A; **2**—AcdA(dT); **3**—AcdA(dT,C11S); **4**—AcdA(dT,C31S); **5**—AcdA(dT,C11S,C31S); **6**—AcdA(N7A); **7**—AcdA(G8A); **8**—AcdA(d9); **9**—AcdA(x4); **10**—OR-7(VI); **11**—avicin A; **12**—AviA(T).

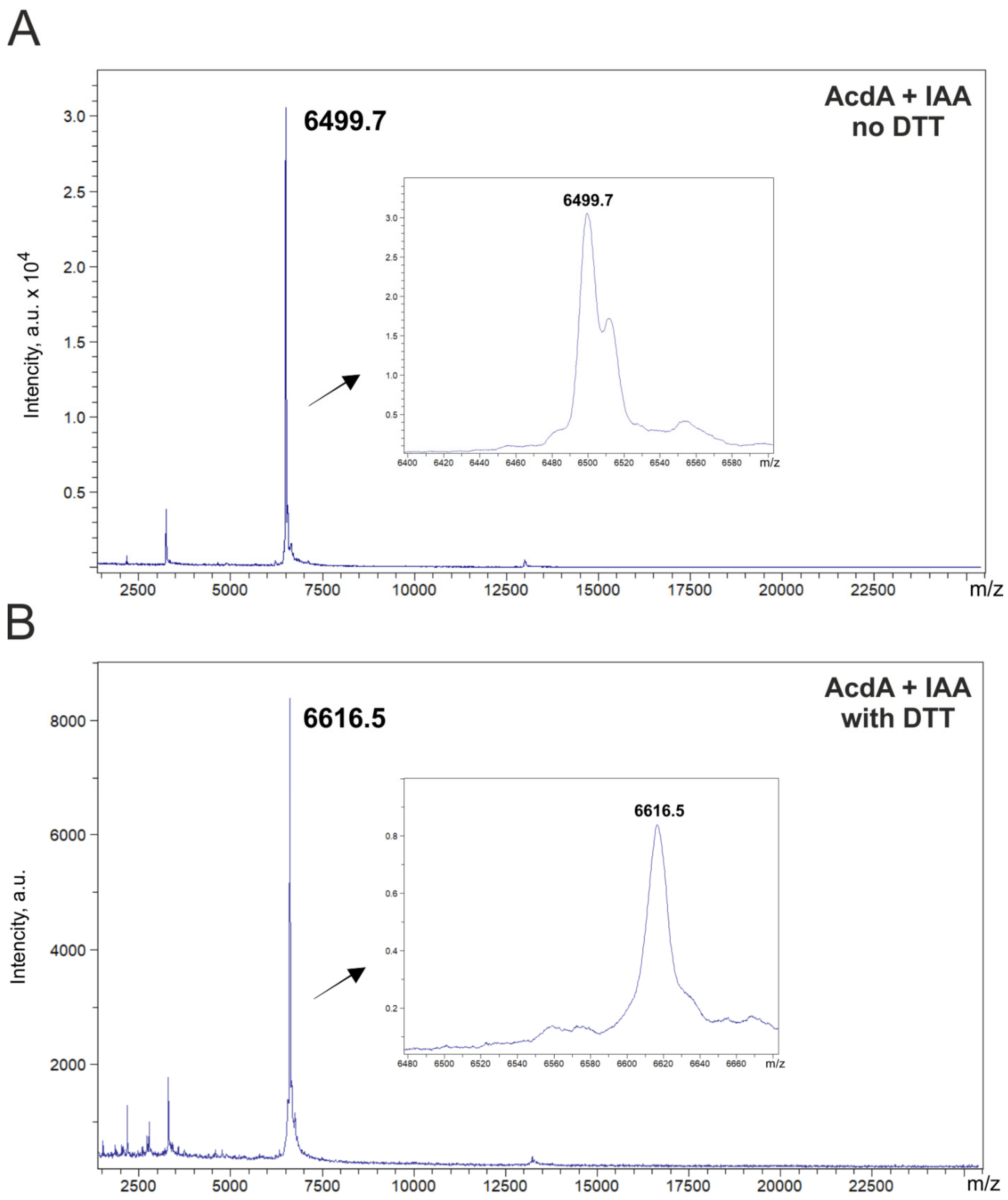

Figure S5. MALDI-TOF mass-spectra of the recombinant acidocin A (AcdA) after incubation with iodoacetamide (IAA) without **(A)** or after **(B)** the addition of 1,4-dithiothreitol (DTT). The difference between the two *m/z* values (117±1 Da) corresponds to two acetamide residues which indicates the absence of free thiol groups in the recombinant peptide.

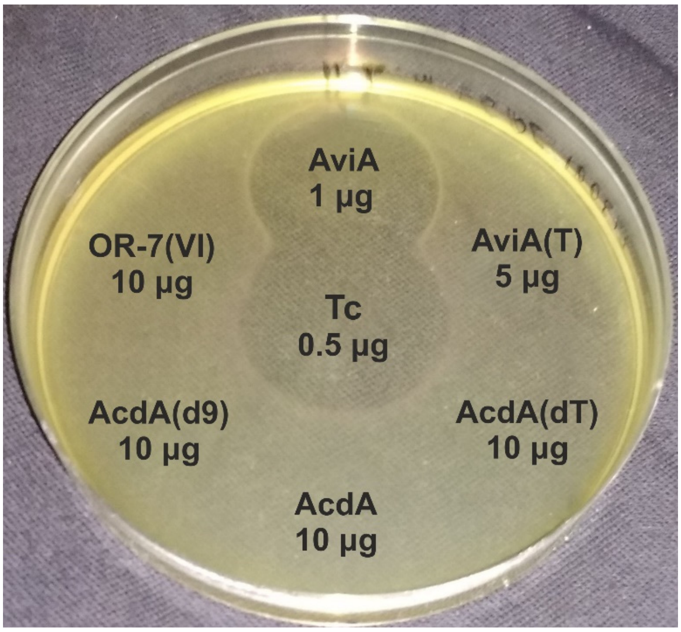

Figure S6. Agar diffusion ("spot-on-lawn") assay of bacteriocins antimicrobial activity against *L. monocytogenes* EGD. 5 µL of bacteriocins/control solution were applied to agar plates containing 3% tryptic soy broth (TSB) medium with mid-log phase culture of *L. monocytogenes* EGD diluted to a OD_{600} of about 0.001 (≅ 7 × 10^5 CFU/mL), and incubated overnight at 37 °C for 24 h. Antibiotic tetracycline (Tc) was used as a positive control.

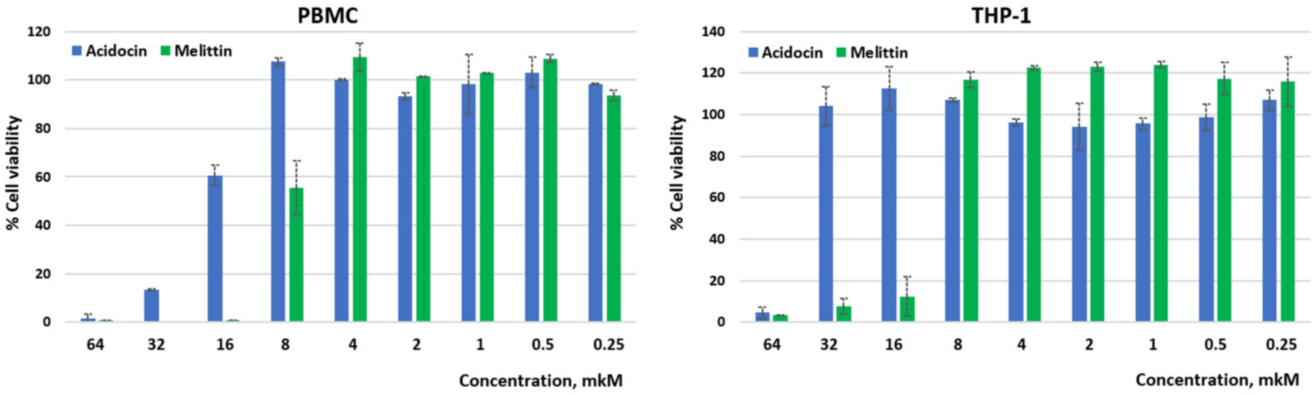

Figure S7. Cytotoxic effects of different concentrations of acidocin A on PBMCs (A) and THP-1 cells (B) after 24 h of incubation. Melittin was used as a positive control. Error bars represent standard deviation between technical replications.

References

1. Orelle, C.; Carlson, S.; Kaushal, B.; Almutairi, M.M.; Liu, H.; Ochabowicz, A.; Quan, S.; Pham, V.C.; Squires, C.L.; Murphy, B.T.; et al. Tools for Characterizing Bacterial Protein Synthesis Inhibitors. *Antimicrob. Agents Chemother.* **2013**, *57*, 5994–6004, doi:10.1128/AAC.01673-13.
